# Dopamine D1 and Glutamate Receptors Co-operate With Brain-Derived Neurotrophic Factor (BDNF) and TrkB to Modulate ERK Signaling in Adult Striatal Slices

**DOI:** 10.3389/fncel.2020.564106

**Published:** 2020-11-16

**Authors:** Ilaria Morella, Harriet Hallum, Riccardo Brambilla

**Affiliations:** ^1^Neuroscience and Mental Health Research Institute, Cardiff University, Cardiff, United Kingdom; ^2^Division of Neuroscience, School of Biosciences, Cardiff University, Cardiff, United Kingdom

**Keywords:** striatum, extracellular-signal regulated kinase (ERK), brain-derived neurotrophic factor (BDNF), dopamine D1 receptors, glutamate receptors

## Abstract

In the striatum, the input nucleus of the basal ganglia, the extracellular-signal-regulated kinase (ERK) pathway, necessary for various forms of behavioral plasticity, is triggered by the combined engagement of dopamine D1 and ionotropic glutamate receptors. In this study, we investigated the potential crosstalk between glutamatergic, dopaminergic, and brain-derived neurotrophic factor (BDNF)-TrkB inputs to ERK cascade by using an *ex vivo* model of mouse striatal slices. Our results confirmed that the concomitant stimulation of D1 and glutamate receptors is necessary to activate ERK in striatal medium spiny neurons (MSNs). Moreover, we found that ERK activation is significantly enhanced when BDNF is co-applied either with glutamate or the D1 agonist SKF38393, supporting the idea of possible integration between BDNF, glutamate, and D1R-mediated signaling. Interestingly, ERK activation *via* BDNF-TrkB is upregulated upon blockade of either AMPAR/NMDAR or D1 receptors, suggesting a negative regulatory action of these two neurotransmitter systems on BDNF-mediated signaling. However, the observed enhancement of ERK1/2 phosphorylation does not result in corresponding downstream signaling changes at the nuclear level. Conversely, the TrkB antagonist cyclotraxin B partially prevents glutamate- and D1-mediated ERK activation. Altogether, these results suggest a complex and unexpected interaction among dopaminergic, glutamatergic, and BDNF receptor systems to modulate the ERK pathway in striatal neurons.

## Introduction

The extracellular-signal regulated kinase (ERK) cascade belongs to the mitogen-activated protein kinase (MAPK) superfamily, a class of highly conserved serine/threonine kinases, originally implicated in cell proliferation, survival, and apoptosis (Mebratu and Tesfaigzi, 2009; Roskoski, 2012). Over the last two decades, considerable attention has been pointed to the multifaceted role of this signaling cascade in a variety of brain functions, from learning and consolidation of long-term memories to drug addiction and neuropsychiatric disorders (Mazzucchelli et al., 2002; Trifilieff et al., 2007; Hart and Balleine, 2016; Krawczyk et al., 2016; Borrie et al., 2017; Morè et al., 2020).

In neuronal cells, a variety of extracellular stimuli such as neurotransmitters, neurotrophic factors, and stressors can trigger the ERK cascade, ultimately leading to the phosphorylation of both cytoplasmic and nuclear factors, including p70 S6 kinase-1 (S6K-1), the MAPK-interacting serine/threonine kinase 1 (Mnk1) and the transcription factors cAMP response element-binding factor (CREB) and Elk-1 (Klann and Dever, 2004; Thomas and Huganir, 2004; Besnard et al., 2011; Wiegert and Bading, 2011; Roskoski, 2012; Yang et al., 2014). Furthermore, ERK1/2 can regulate gene transcription by phosphorylating histone H3 on Ser10 *via* the kinases MSK-1 and MSK-2 (Brami-Cherrier et al., 2005; Santini et al., 2007).

In the striatum, the input nucleus of the basal ganglia, the ERK cascade acts as a coincident detector between the dopaminergic and glutamatergic pathways. More specifically, glutamate-induced ERK activation is amplified by D1 receptors stimulation *via* cAMP, PKA, and cAMP-regulated phosphoprotein (DARPP-32) pathway, which maintains ERK1/2 in their active forms *via* the concerted inhibitory action of striatal-enriched tyrosine phosphatase (STEP) and protein phosphatase 1 (PP1; Valjent et al., 2005; Pascoli et al., 2011). This ERK regulation occurs in the medium spiny neurons of the direct striatal pathway (dMSNs), where D1Rs are primarily expressed. The same striatal pathway is also implicated in the responses to drugs of abuse such as cocaine and amphetamine (Nestler and Lüscher, 2019). However, very little is known of the role of ERK signaling modulation in the medium spiny neuron’s indirect striatal pathway (iMSNs).

Besides the indirect crosstalk between NMDARs and dopamine D1 receptors in dMSNs, other studies have found physical interactions between the C-terminal tails of D1Rs with GluN1 NMDAR subunit that enable an increased membrane insertion of D1Rs and are also implicated in the attenuation of NMDAR-dependent excitotoxicity through a PI-3 kinase-dependent pathway (Pei et al., 2004; Cahill et al., 2014a). Also, It has been postulated that the integration of dopaminergic and glutamatergic stimuli in the direct striatal pathway occurs *via* Ras-GRF1 and Ras-GRF2, a family of Ras exchange factor specifically expressed in the central nervous system, also necessary for cocaine-induced ERK activation and long-term behavioral changes (Fasano et al., 2009; Bernardi et al., 2019).

Moreover, it is well established that the ERK pathway is one of the molecular effectors of the brain-derived neurotrophic factor (BDNF). The mature form of BDNF binds to the tropomyosin receptor kinase B (TrkB), thus activating not only the Ras-ERK cascade but also the phosphatidylinositol-3-kinase (PI3 kinase)/Akt and phospholipase Cγ (PLCγ) pathways. Crucially, BDNF-TrkB mediated signaling has been linked to synaptogenesis and synaptic plasticity mechanisms underlying learning and memory (Cunha et al., 2010; Park and Poo, 2013).

In the striatum, BDNF-TrkB-ERK signaling is believed to exert a primary pro-survival role for all striatal MSNs, both belonging to the direct and the indirect pathway (Nagahara and Tuszynski, 2011; Baydyuk and Xu, 2014). However, circumstantial evidence has also positively implicated BDNF-TrkB signaling in behavioral and cellular plasticity produced by drugs of abuse, including cocaine (McGinty et al., 2010; Koo et al., 2012; Li et al., 2013) and amphetamine (McGinty et al., 2011), which both require the concerted action of dopaminergic and glutamatergic signaling in dMSNs. However, the crosstalk between dopaminergic/glutamatergic and BDNF signaling is poorly understood in dMSNs and their effect on ERK modulation remains to be explored.

Our results, using an *ex vivo*, adult mouse striatal slice stimulation system, suggest that a complex integration between glutamatergic, dopaminergic, and BDNF inputs may occur in striatal neurons and converges onto the ERK cascade.

## Materials and Methods

### Animals

Two months old C57BL/6 male mice were purchased from Charles River and housed in a temperature-controlled (21°C) environment maintained on a 12-h light–dark cycle. Mice were given 2–3 days acclimatization period in their holding cages with food and water available *ad libitum* before being sacrificed by cervical dislocation. Animal experiments adhered to the UK Home Office Animals (Scientific Procedures) Act 1986 and European Community guidelines (*Directive 2010*/63/*EU)* on animal experimentation. In total, four mice were used for each dose-response experiment and eight mice were used for each of the other studies. All experiments were performed in duplicate.

### *Ex vivo* Brain Slices Preparation

Brains slices were prepared as previously described (Cerovic et al., 2015; Papale et al., 2016). Brains were rapidly removed from the skull, mounted onto the vibratome stage (Vibratome, VT1000S-Leica Microsystems), and cut into 200 μm-thick coronal slices. The slices were taken across the striatum (from AP = 1.18 mm and AP = 0.02 mm) and randomly assigned to the different treatments. During the cutting, brains were kept submerged in ice-cold sucrose-based dissecting solution (87 mM NaCl, 2.5 mM KCl, 7 mM MgCl_2_, 1 mM NaH_2_PO_4_, 75 mM sucrose, 25 mM NaHCO_3_, 10 mM D -glucose, 0.5 mM CaCl_2_, 2 mM kynurenic acid), oxygenated with 95% O_2_ and 5% CO_2_. Corticostriatal slices were subsequently transferred into a brain slice chamber (Brain slice chamber-BSC1—Scientific System Design Inc., Mississauga, ON, Canada) and allowed to recover for 1 h at 32°C, with a constant perfusion of carboxygenated artificial cerebrospinal fluid (ACSF: 124 mM NaCl, 5 mM KCl, 1.3 mM MgSO_4_7H_2_O, 1.2 mM NaH_2_PO_4_H_2_O, 2.5 mM NaHCO_3_, 10 mM glucose, 2.4 mM CaCl_2_). SCH233390, CNQX, AP5, MPEP, LY367385, and cyclotraxin B (Tocris) were applied to the slices at the doses indicated for 1 h during the recovery period, whereas SKF38393 (Sigma–Aldrich), BDNF (Tocris), or glutamate (Sigma–Aldrich) were applied for 10 min after the recovery period at the doses indicated.

Following a brief fixation in 4% PFA for 15 min at room temperature, the slices were rinsed in PBS and cryoprotected in 30% sucrose solution overnight at 4°C. On the following day, the slices were further cut into 18 μm-thick sections using a cryostat (Leica CM1850), mounted onto SuperFrost Plus slides (Thermo Scientific), and processed for immunohistochemistry or immunofluorescence.

### Immunohistochemistry

Immunohistochemistry was performed as described in Papale et al. (2016). After quenching with 3% H_2_O_2_, 10% methanol for 15 min, the sections were rinsed in TBS and incubated for 1 h in blocking solution (5% normal goat serum, 0.1% Triton X-100). The sections were then incubated overnight at 4°C with anti-phospho-p44/42 MAP kinase (Thr202/Tyr204; 1:1,000, Cell Signaling Technology, Danvers, MA, USA). On the following day, a biotinylated goat anti-rabbit secondary antibody (1:200, Vector Labs) was applied to the sections for 2 h at room temperature. Detection of the bound antibodies was carried out using a standard peroxidase-based method (ABC-kit, Vectastain, Vector Labs), followed by incubation with DAB and H_2_O_2_ solution. The sections were subsequently dehydrated using increasing concentrations of ethanol and mounted with DPX. Images were acquired from the striatum using a bright-field microscope (Leica DMI6000B Macro/Microimaging system) under a 20× magnification.

### Immunofluorescence

Immunofluorescence was performed as described in Papale et al. (2016). One hour after blocking in 5% normal goat serum and 0.1% Triton X-100 solution, the slices were incubated overnight at 4°C with one of the following primary antibodies: anti-phospho-S6 ribosomal protein (Ser235/236; 1:200, Cell Signaling Technology, Danvers, MA, USA) or anti-phospho- (Ser10)-acetylated (Lys14) histone H3 (1:1,000, Millipore, Billerica, MA, USA) and anti-NeuN (1:1,000, Millipore, Billerica, MA, USA). The sections were then incubated for 1 h at room temperature with the following secondary antibodies: AlexaFluor 546 conjugated anti-mouse (1:200, Life Technologies) and AlexaFluor 488 conjugated anti-rabbit (1:500, Life Technologies). Single and double-labeled images (1,024 × 1,024 μm) were obtained at 40× magnification from striatum using a laser scanning confocal microscopy (Leica SP2) equipped with the corresponding lasers and appropriate filters sets to avoid the crosstalk between the fluorochromes.

### Image Quantification and Statistics

Neuronal quantification was performed using ImageJ software.

For immunohistochemistry experiments, data are expressed as the average number of phospho-ERK positive cells counted in four fields per slice in 2–3 consecutive 18 μm-thick slices (size of field: 0.036 mm^2^).

For immunofluorescence experiments, data are presented as the average number of phospho-S6 or phospho-Ac-H3 positive cells among NeuN positive cells counted in six fields per slice in 2–3 consecutive 18 μm-thick slices.

Statistical analysis was performed with IBM SPSS Statistics software (version 25). For dose-response experiments one-way ANOVAs or Kruskal–Wallis tests were conducted, whereas two-way ANOVAs were carried out for co-administration of compounds. For each experimental group, the number of samples is given by the number of 200 μm-thick slices.

Dose-response curves were generated by GraphPad Prism software (version 8.3.1). The number of pERK1/2 positive cells was normalized over not stimulated controls and reported on Y-axis. The doses of glutamate, SKF38393, and BDNF are shown on X-axis in a logarithmic (Log10) scale. The EC50 values were calculated by GraphPad Prism using a non-linear regression model.

## Results

### Glutamate, the D1 Receptor Agonist SKF38393, and BDNF Independently Lead to a Rapid Dose-Dependent ERK Phosphorylation in Striatal Slices

Previous work has shown that ERK activation in the striatal network is a central integration point for ionotropic glutamate receptors (AMPARs and NMDARs) and dopamine D1 receptors signaling (Cahill et al., 2014b; Pascoli et al., 2014). However, the potential interplay between glutamate and dopamine signaling with BDNF and TrkB receptors in striatal preparations has not been fully explored. Thus, we sought to carefully investigate the potential interaction among AMPARs/NMDARs, D1Rs, and TrkB receptors in a physiological setting consisting of *ex vivo* striatal slices preparations from the adult mouse brain (Cerovic et al., 2015; Papale et al., 2016). This system is better suited to represent an *in vivo* situation than embryonic or early postnatal organotypic cultures and also allows single-cell analysis which is precluded by standard western blot analysis.

First, we determined the minimal effective doses of glutamate, of the selective D1 agonist SKF38393 and of BDNF sufficient to phosphorylate ERK1/2, the downstream cytoplasmic marker S6 ribosomal protein, and the nuclear marker histone H3 in mouse corticostriatal slices. This experiment was important to demonstrate whether all three stimuli could activate ERK signaling in the cytoplasm and in the nucleus and to determine the doses to be used in co-stimulation experiments.

**Figure 1:**
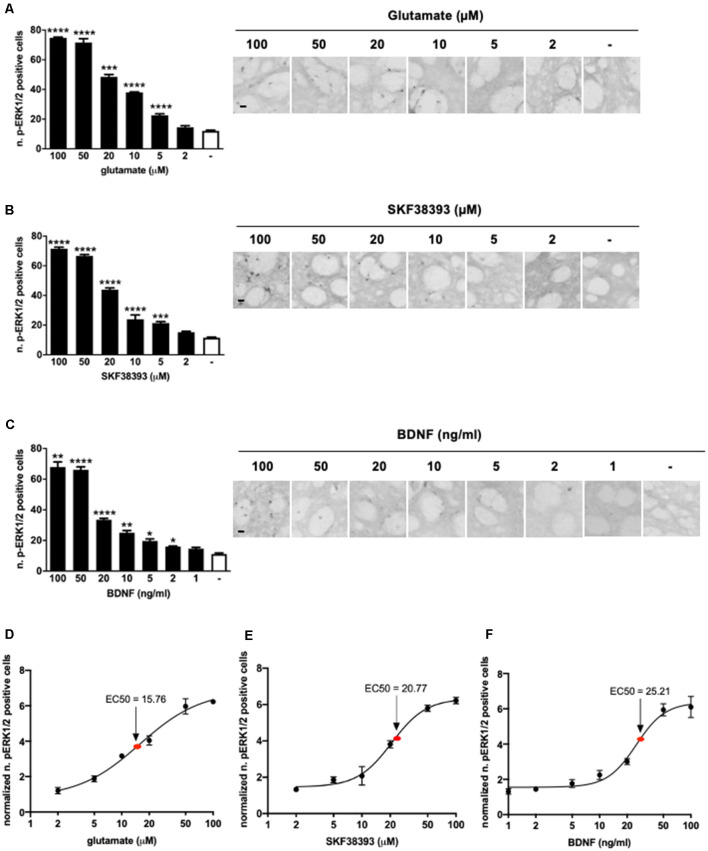
**(A)** Glutamate induces significant extracellular-signal-regulated kinase (ERK) activation at doses ranging from 5 μM to 100 μM (one-way ANOVA, Welch’s *F*_(6,9.064)_ = 1,001.854, *p* < 0.0001. Games Howell’s *post hoc*: comparisons were made between the not stimulated condition and each glutamate dose: ****p < 0.0001, ***p < 0.001). Data are shown as mean ± SEM, *n* = 4 per group. Scale bar: 50 μm. **(B)** SKF38393 activates ERK at doses ranging from 5 μM to 100 μM (one-way ANOVA, *F*_(6,27)_ = 320.491, *p* < 0.0001. Bonferroni’s *post hoc*: comparisons were made between the not stimulated condition and each SKF38393 dose: ****p < 0.0001, ***p < 0.001). Data are shown as mean ± SEM, *n* = 4 per group. Scale bar: 50 μm. **(C)** Brain-derived neurotrophic factor (BDNF) activates ERK at doses ranging from 2 ng/ml to 100 ng/ml (one-way ANOVA, Welch’s *F*_(7,9.911)_ = 134.027, *p* < 0.0001. Games Howell’s *post hoc*: comparisons were made between the not stimulated condition and each BDNF dose: ****p < 0.0001, **p < 0.01, *p < 0.05). Data are shown as mean ± SEM, *n* = 4 per group. Scale bar: 50 μm. **(D–F)** Dose-response curves of glutamate, SKF38393, and BDNF on ERK activation. Doses are reported on X-axis in a logarithmic (Log10) scale, whereas Y-axis shows the number of pERK1/2 positive cells normalized over not stimulated controls. The EC50 values were calculated using GraphPad Prism software.

Samples were stimulated for 10 min with increasing concentrations of glutamate, SKF38393, or BDNF and, after fixation, were processed for immunohistochemistry to detect phospho-ERK1/2 positive cells in the striatum. Data indicated that 5 μM of glutamate (Figure 1A), 5 μM of SKF38393 (Figure 1B), and 2 ng/ml of BDNF (Figure 1C) were sufficient to induce a significant increase in ERK1/2 activation, whereas maximal ERK1/2 activation was achieved at 50 μM for all three stimuli.

Dose-response curves (Figures 1D–F) indicated that the EC50 was 15.76 μM for glutamate, 20.77 μM for SKF38293, and 25.21 ng/ml for BDNF. Thus, for further co-stimulation experiments, we selected sub-optimal doses just below the EC50, namely 10 μM for glutamate, 10 μM for SKF38393, and 10 ng/ml for BDNF.

### Glutamate, SKF38393, and BDNF Rapidly Phosphorylate Striatal S6 Ribosomal Protein and Histone H3 in an ERK-Dependent Manner

To confirm that all three stimuli were able to activate downstream signaling to ERK1/2, striatal slices were also subjected to immunofluorescence analysis to visualize S6 phosphorylation, assessed at Ser235/236 sites, and histone H3 phosphorylation at the Ser10 site (and acetylation at Lys14). Our previous work has shown that those two phosphorylation sites are entirely ERK1/2 dependent in response to glutamate and SKF38393 (Papale et al., 2016). However, we never determined whether BDNF was able to induce S6 and H3 at those phosphorylation sites.

**Figure 2:**
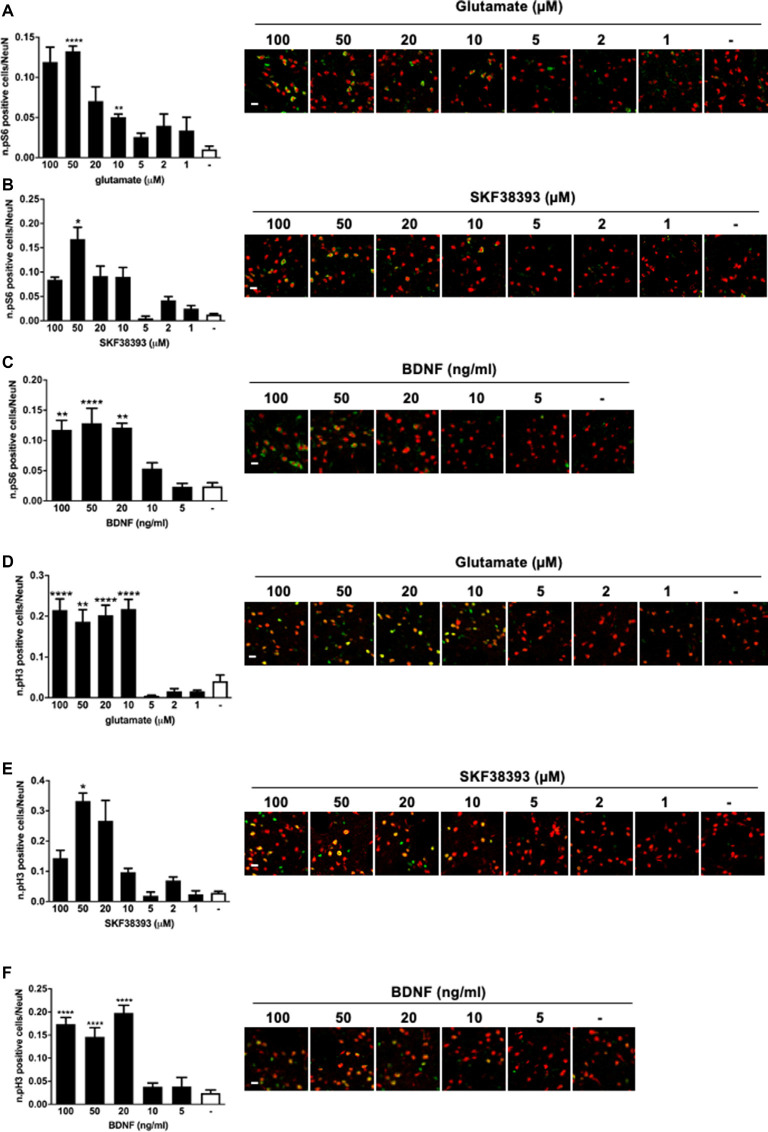
**(A)** Glutamate induces phospho-S6 activation at the doses of 50 and 10 μM (one-way ANOVA, Welch’s F *F*_(7,10.078)_ = 29.488, *p* < 0.0001. Games Howell’s *post hoc*: not stimulated vs. 50 μM ****p < 0.0001, not stimulated vs. 10 μM **p < 0.01). Scale bar: 50 μm. **(B)** SKF38393 50 μM activates S6 (Kruskal–Wallis test, *H*_(7)_ = 26.532, *p* < 0.0001. Pairwise comparisons: not stimulated vs. 50 μM, *p < 0.05). Scale bar: 50 μm. **(C)** BDNF activates S6 at doses ranging from 20 ng/ml to 100 ng/ml (one-way ANOVA, *F*_(5,23)_ = 14.074, *p* < 0.0001. Bonferroni’s *post hoc*: not stimulated vs. 100 ng/ml **p < 0.01, not stimulated vs. 50 ng/ml ****p < 0.0001, not stimulated vs. 20 ng/ml **p < 0.01). Scale bar: 50 μm. **(D)** Glutamate induces phospho-H3 at doses ranging from 10 to 100 μM (one-way ANOVA, Welch’s *F*_(7,9.401)_ = 25.373, *p* < 0.0001. Bonferroni’s *post hoc*: comparisons were made between the not stimulated condition and each glutamate dose: ****p < 0.0001, **p < 0.01). Scale bar: 50 μm. **(E)** H3 is phosphorylated by SKF38393 50 μM (Kruskal–Wallis test, *H*_(7)_ = 27.273, *p* < 0.0001. Pairwise comparisons: not stimulated vs. 50 μM, *p < 0.05). Scale bar: 50 μm. **(F)** BDNF activated H3 at doses ranging from 20 to 100 ng/ml. One-way ANOVA, *F*_(5,23)_ = 26.854, *p* < 0.0001. Bonferroni’s *post hoc* between not stimulated conditions and each BDNF dose: ****p < 0.0001). Scale bar: 50 μm. In all graphs, data are shown as mean ± SEM, *n* = 4 per group. In the representative pictures, the red channel (NeuN) is merged with the green channel (phospho-H3 or phospho-S6).

We found that all three stimuli could activate pS6 and pH3 but with remarkable differences. In general, higher doses were required to elicit S6 and H3 phosphorylation than for ERK1/2. More specifically, 50 μM of glutamate, 50 μM of SKF38393, and 20 ng/ml of BDNF induced a maximal increase of both S6 (Figures 2A–C) and H3 phosphorylation (Figures 2D–F), whereas lower doses failed to elicit a significant activation of both S6 and H3. Thus, it appears that phosphorylation of S6 and H3 requires a higher concentration of each agonist than for ERK1/2 activation and this is possibly subjected to a threshold effect. Since we were unable to determine with accuracy an EC50 for either pS6 or pH3, we focussed exclusively on pERK1/2 analysis in the subsequent co-stimulation studies.

### Co-stimulation With Glutamate and SKF38393 Enhances ERK1/2 Activation

Early evidence showed that cocaine-induced ERK activation *in vivo* in the striatum requires the concomitant stimulation of D1 and NMDA receptors (Valjent et al., 2005). Also, crosstalk between D1 and NMDA receptors is necessary to activate ERK in organotypic slices (Fasano et al., 2009), and that GluN1 and D1 receptor may physically interact to achieve full activation of downstream ERK signaling (Pascoli et al., 2011; Cahill et al., 2014a).

**Figure 3:**
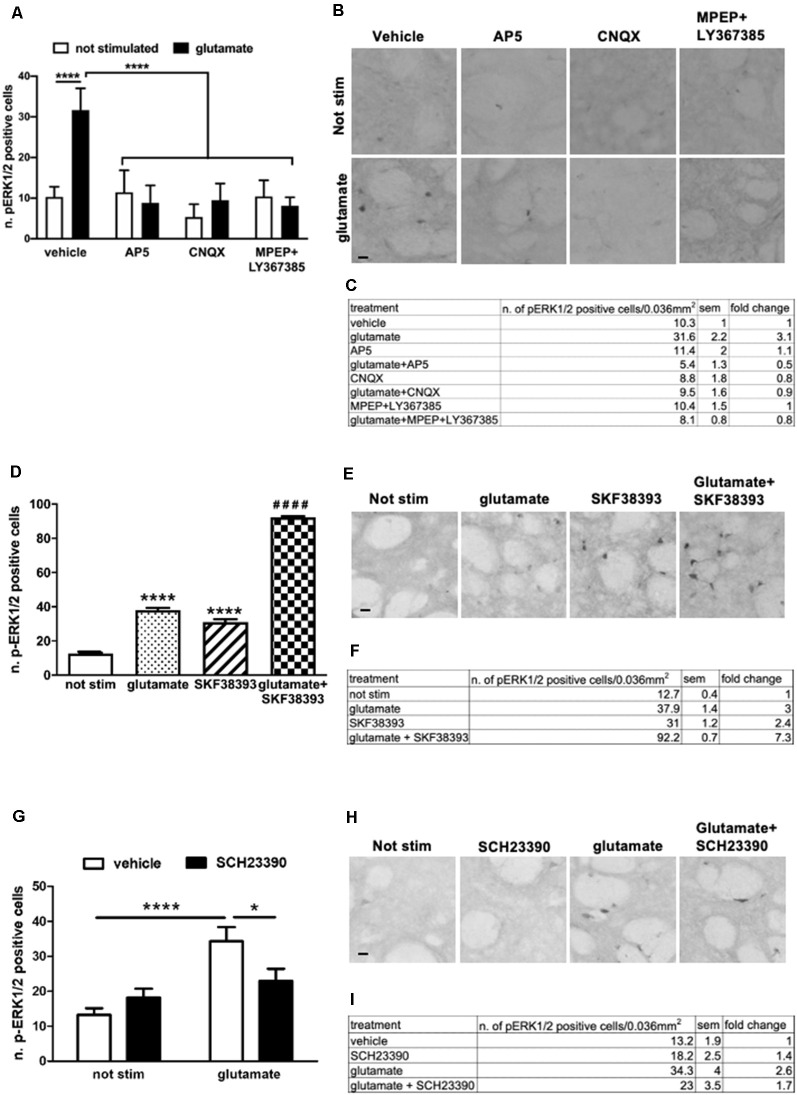
**(A,C)** Pre-treatment with AP5 10 μM, CNQX 10 μM or with a mix of MPEP 20 μM and LY367385 100 μM completely prevents glutamate-induced ERK activation (two-way ANOVA, effect of glutamate *F*_(1,43)_ = 20.74, *p* < 0.0001, effect of inhibitors *F*_(3,43)_ = 28.08, *p* < 0.0001, effect of interaction *F*_(3,43)_ = 24.24, *p* < 0.0001. Bonferroni’s *post hoc*, not stimulated vs. glutamate ****p < 0.0001, AP5 + glu vs. glu ****p < 0.0001, CNQX + glu vs. glu ****p < 0.0001, MPEP + Ly367385 + glu vs. glu ****p < 0.0001). The values of mean, SEM and fold change relative to not stimulated vehicle are shown in panel **(C)**. Data are shown as mean ± SEM. **(B)** Representative pictures of anti-phospho-ERK1/2 immunohistochemistry. Scale bar: 50 μm. **(D,F)** Slices co-stimulated with SKF38393 10 μM and glutamate 10 μM show increased levels of phospho-ERK1/2 in comparison with those stimulated with SKF38393 10 μM or glutamate 10 μM (two-way ANOVA, effect of glutamate *F*_(1,24)_ = 1759.699 *p* < 0.0001, effect of SKF38393 *F*_(1,24)_ = 1247.512, *p* < 0.0001, effect of interaction *F*_(1,24)_ = 305.606, *p* < 0.0001. Bonferroni’s *post hoc*, not stimulated vs. glutamate: ****p < 0.0001, not stimulated vs. SKF38393: ****p < 0.0001, SKF38393 vs. SKF38393 + glutamate: ^####^*p* < 0.0001, glutamate vs. SKF38393 + glutamate: ^####^*p* < 0.0001). Data are shown as mean ± SEM. The values of mean, SEM and fold change relative to not stimulated control are shown in panel **(F)**. Not stimulated: *n* = 8, glutamate: *n* = 6, SKF38393: *n* = 6, SKF38393 + glutamate: *n* = 8. **(E)** Representative pictures of anti-phospho-ERK1/2 immunohistochemistry. Scale bar: 50 μm. **(G,I)** Pre-treatment with the D1 antagonist SCH23390 20 μM prevents glutamate-induced ERK activation (two-way ANOVA, effect of glutamate *F*_(1,29)_ = 18.343, *p* < 0.0001, effect of SCH23390 *F*_(1,29)_ = 1.147, *p* = 0.294, effect of interaction *F*_(1,29)_ = 7.342, *p* = 0.012. Bonferroni’s *post hoc*, not stimulated vs. glutamate: ****p < 0.0001, glutamate vs. glutamate + SCH23390: *p = 0.016). Data are shown as mean ± SEM. The values of mean, SEM and fold change relative to vehicle control are shown in panel **(I)**. Not stimulated: *n* = 8, SCH23390: *n* = 8, glutamate: *n* = 7, glutamate + SCH23390: *n* = 7. **(H)** Representative pictures of anti-phospho-ERK1/2 immunohistochemistry. Scale bar: 50 μm.

To further confirm and extend these observations in our *ex vivo* model, we first assessed the contribution of NMDA, AMPA, and metabotropic receptors in glutamate-induced ERK activation. In particular, we focused on the group I mGluR1 and mGluR5. This class of receptors is coupled to Gq and, in response to glutamate, stimulates the phospholipase C pathway. Importantly, mGlu1/5 has been shown to activate the ERK cascade which in turn regulates mGluR1/5 signaling and functions (Mao and Wang, 2016). First, we pre-treated the striatal slices for 1 h with the NMDAR antagonist (2*R*)-amino-5-phosphonovaleric acid (AP5, 10 μM), the AMPAR antagonist cyanquixaline (CNQX, 10 μM), or with a cocktail of mGlu1 and mGlu5 antagonists, respectively LY367385 (100 μM) and 6-Methyl-2-(phenylethynyl)pyridine hydrochloride (MPEP, 20 μM). Next, we stimulated the slices for 10 min with a sub-optimal dose of glutamate (10 μM). Interestingly, we demonstrated that glutamate-induced ERK activation is completely prevented in the presence of either NMDA, AMPA, and mGlu1/5 receptors antagonists (Figures 3A–C).

Next, we treated slices with sub-optimal doses of glutamate (10 μM) and SKF38393 (10 μM), alone or in combination. As shown in Figures 3D–F, the concomitant stimulation of glutamate and D1 receptors further enhanced ERK phosphorylation. The activation is not simply additive but possibly there is a synergistic component. The co-stimulation induced a 138% fold increase in ERK activation over glutamate and SKF38393 alone (Figure 3F).

Moreover, a pre-treatment with the D1 antagonist SCH23390 (20 μM) significantly attenuated glutamate-induced ERK1/2 activation, thus indicating that intact D1-mediated signaling is required for glutamate to trigger ERK signaling (Figures 3G–I). These data confirmed and extended our previous data on striatal organotypic cultures which support the idea that glutamate and D1 receptors interact functionally to fully induce ERK activation.

### BDNF-Mediated ERK Activation Is Enhanced by a Co-stimulation With Either SKF38393 or Glutamate

Potential interactions between dopamine D1 receptors and TrkB receptors or between glutamate receptors and TrkB receptors have never been investigated in adult striatal systems, although some aspects of BDNF signaling have been studied in striatal cultures (Gokce et al., 2009). This is an important issue since our previous work indicated that BDNF-mediated ERK activation in striatal organotypic cultures is Ras-GRF1 independent (Fasano et al., 2009). Since Ras-GRF1 is a crucial, striatal enriched signaling integrator of both NMDARs and D1Rs on ERK, it is conceivable that BNDF ability to activate ERK may be independent of the other two neurotransmitter systems.

**Figure 4:**
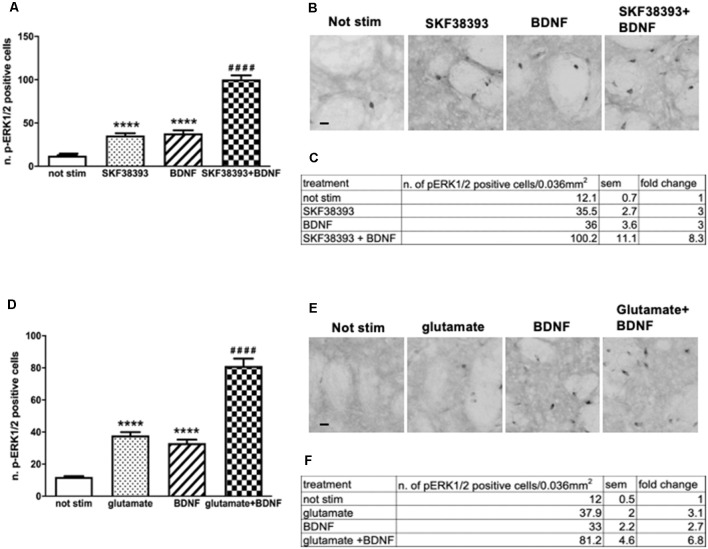
**(A,C)** Slices co-stimulated with BDNF 10 ng/ml and SKF38393 10 μM show increased levels of phospho-ERK1/2 (two-way ANOVA, effect of SKF38393 *F*_(1,26)_ = 176.023 *p* < 0.0001, effect of BDNF *F*_(1,26)_ = 180.356 *p* < 0.0001, effect of interaction *F*_(1,26)_ = 38.267 *p* < 0.0001, Bonferroni’s *post hoc*: not stimulated vs. BDNF: ****p < 0.0001, not stimulated vs. SKF38393: ****p < 0.001, SKF38393 vs. BDNF + SKF38393: ^####^*p* < 0.0001, BDNF vs. BDNF + SKF38393: ^####^*p* < 0.0001). Data are shown as mean ± SEM. The values of mean, SEM and fold change relative to not stimulated control are shown in panel **(C)**. Not stimulated: *n* = 8, SKF38393: *n* = 7, BDNF: *n* = 7, BDNF + SKF38393: *n* = 8. **(B)** Representative pictures of anti-phospho-ERK1/2 immunohistochemistry. Scale bar: 50 μm. **(D,F)** Slices co-stimulated with BDNF 10 ng/ml and glutamate 10 μM show enhanced ERK activation (two-way ANOVA, effect of glutamate *F*_(1,24)_ = 154.64 *p* < 0.0001, effect of BDNF *F*_(1,24)_ = 116.887 *p* < 0.0001, effect of interaction *F*_(1,24)_ = 13.999 *p* = 0.001. Bonferroni’s *post hoc*, not stimulated vs. BDNF: ****p < 0.0001, not stimulated vs. glutamate: ****p < 0.001, glutamate vs. BDNF + glutamate: ^####^*p* < 0.0001, BDNF vs. BDNF + glutamate: ^####^*p* < 0.0001). Data are shown as mean ± SEM. The values of mean, SEM and fold change relative to vehicle control are shown in panel **(F)**. Not stimulated : *n* = 8, glutamate: *n* = 6, BDNF: *n* = 6, glutamate + BDNF: *n* = 8. **(E)** Representative pictures of anti-phospho-ERK1/2 immunohistochemistry. Scale bar: 50 μm.

Firstly, to determine whether potential crosstalk between BDNF and D1-mediated signaling exists, striatal slices were treated with 10 ng/ml BDNF, 10 μM SKF38393 or co-stimulated with 10 ng/ml BDNF and 10 μM SKF38393 for 10 min. As shown in Figures 4A,B, the co-application of BDNF and SKF38393 has a partial synergistic effect on ERK phosphorylation, with a 138% fold increase in ERK activation upon co-stimulation over BDNF and SKF38393 alone (Figure 4C).

Similarly, we sought to determine the potential interaction between BDNF- and glutamate-mediated signaling by co-stimulating striatal slices with 10 ng/ml BDNF and 10 μM glutamate for 10 min. Our results demonstrated that BDNF-mediated ERK phosphorylation is further enhanced by glutamate (Figures 4D,E). Somewhat, BDNF-glutamate interactions appeared to be less pronounced, with a 119% fold increase in ERK activation upon co-stimulation over BDNF and glutamate alone (Figure 4F).

Altogether, the data indicate that the co-stimulation of BDNF with the D1R agonist or glutamate may lead to some functional interactions between the two systems converging on ERK signaling.

### BDNF-Induced ERK Activation Is Enhanced Upon Either D1R- or AMPAR/NMDAR-Mediated Signaling Blockade

To determine whether BDNF-TrkB receptors require either D1 receptors, glutamate receptors, or both to induce maximal ERK activation, we pre-treated the slices for 1 h with the D1R antagonist SCH23390 (20 μM) or with a cocktail of CNQX (10 μM) and AP5 (10 μM). If ERK activation induced by BDNF-TrkB receptors is independent of the dopaminergic and glutamatergic systems, it should also be insensitive to their receptors blockade.

**Figure 5:**
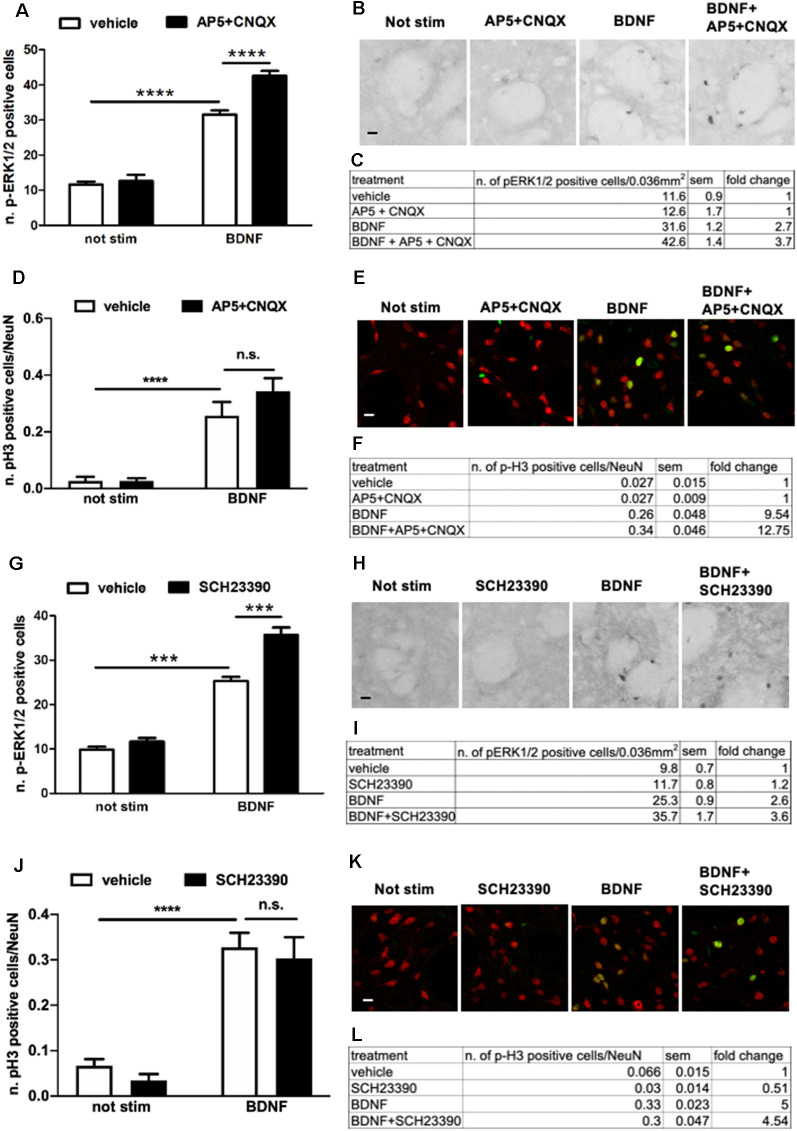
**(A,C)** Pre-treatment with glutamate antagonists CNQX 10 μM and AP5 10 μM increases BDNF-induced ERK activation (two-way ANOVA, effect of BDNF *F*_(1,28)_ = 341.384, *p* < 0.0001, effect of AP5/CNQX *F*_(1,28)_ = 19.969, *p* < 0.0001, effect of interaction *F*_(1,28)_ = 13.553, *p* = 0.001. Bonferroni’s *post hoc*, not stimulated vs. BDNF: ****p < 0.0001, BDNF vs. BDNF + AP5 + CNQX: ****p < 0.0001). Data are shown as mean ± SEM, *n* = 8 per group. The values of mean, SEM and fold change relative to not stimulated control are shown in panel **(C)**. **(B)** Representative pictures of anti-phospho-ERK1/2 immunohistochemistry. Scale bar: 50 μm. **(D,F)** Pre-treatment with glutamate antagonists CNQX 10 μM and AP5 10 μM has no effect on BDNF-induced H3 activation (two-way ANOVA, effect of BDNF *F*_(1,23)_ = 50.80, *p* < 0.0001, effect of AP5/CNQX *F*_(1,23)_ = 1.272, *p* = 0.2710, effect of interaction *F*_(1,23)_ = 1.279, *p* = 0.2697. Bonferroni’s *post hoc*, not stimulated vs. BDNF ***p = 0.0007, AP5 + CNQX vs. BDNF + AP5 + CNQX ****p < 0.0001, BDNF vs. BDNF + AP5 + CNQX *p* = 0.2082). Data are shown as mean ± SEM. Not stimulated and AP5 + CNQX: *n* = 6 per group, BDNF: *n* = 7, BDNF + AP5 + CNQX: *n* = 8. The values of mean, SEM, and fold change relative to not stimulated vehicles are shown in panel **(F)**; n.s.: not significant. **(E)** Representative pictures of phospho-H3 immunofluorescence, where the red channel (NeuN) is merged with the green channel (phospho-H3). Scale bar: 50 μm. **(G,I)** Pre-treatment with D1-antagonist SCH23390 upregulates BDNF-induced ERK phosphorylation (two-way ANOVA, effect of BDNF *F*_(1,28)_ = 331.98, *p* < 0.0001, effect of SCH23390 *F*_(1,28)_ = 31.82, *p* < 0.0001, effect of interaction *F*_(1,28)_ = 15.51, *p* = 0.0005. Bonferroni’s *post hoc*, not stimulated vs. BDNF ***p < 0.001, BDNF vs. BDNF + SCH23390 ***p < 0.001). Data are shown as mean ± SEM, *n* = 8 per group. The values of mean, SEM, and fold change relative to not stimulated control are shown in panel **(I)**. **(H)** Representative pictures of anti-phospho-ERK1/2 immunohistochemistry. Scale bar: 50 μm. **(J,L)** Pre-treatment with D1-antagonist SCH23390 does not affect BDNF-induced H3 phosphorylation (two-way ANOVA, effect of BDNF *F*_(1,28)_ = 76.97, *p* < 0.0001, effect of SCH23390 *F*_(1,28)_ = 0.8778, *p* = 0.3568, effect of interaction *F*_(1,28)_ = 0.01749, *p* = 0.8957. Bonferroni’s *post hoc*, not stimulated vs. BDNF ****p < 0.0001, SCH23390 vs. BDNF + SCH23390 ****p < 0.0001, BDNF vs. BDNF + SCH23390 *p* > 0.9999). Data are shown as mean ± SEM, *n* = 8 per group. The values of mean, SEM, and fold change relative to not stimulated vehicles are shown in panel **(L)**; n.s.: not significant. **(K)** Representative pictures of phospho-H3 immunofluorescence, where the red channel (NeuN) is merged with the green channel (phospho-H3). Scale bar: 50 μm.

Surprisingly, we found that upon BDNF stimulation (10 ng/ml, for 10 min) ERK1/2 phosphorylation was significantly enhanced in the presence of either AMPAR/NMDAR (Figures 5A–C) or D1R antagonists (Figures 5G–I). In both cases, the effect was highly significant, with a 137% and 138% fold increase in ERK activation upon AMPAR/NMDAR and D1R blockade, respectively, over BDNF alone (Figures 5C,I).

To verify whether the modulation of BDNF signaling by D1R and AMPAR/NMDAR results in downstream changes at the nuclear level, we measured histone H3 phosphorylation upon BDNF stimulation in the presence of either AMPAR/NMDAR and D1R antagonists. Since we previously found that BDNF at a dose of 10 ng/ml used for the previous co-stimulation studies was insufficient to activate histone H3 (Figure 2F), in the present experiment we stimulated slices with BDNF 20 ng/ml which significantly enhances pH3. We found that BDNF-induced histone H3 phosphorylation was not further upregulated by either AMPA/NMDA (Figures 5D–F) or D1 receptors blockade (Figures 5J–L).

Overall, these data revealed an unexpected complexity of the modulation of BDNF-TrkB-ERK signaling by AMPARs/NMDARs and D1 receptors.

### Pre-treatment With TrkB Antagonist Cyclotraxin B Reduces Glutamate- and SKF38393-Induced ERK Activation

TrkB-mediated ERK activation depends on the simultaneous engagement of D1 and glutamatergic systems, suggesting a competition between BDNF-TrkB on one side and glutamate and D1 receptors on the other. In principle, such interaction should be reciprocal, i.e., TrkB receptor blockade should facilitate glutamate and D1 receptors positive modulation on ERK activity.

**Figure 6:**
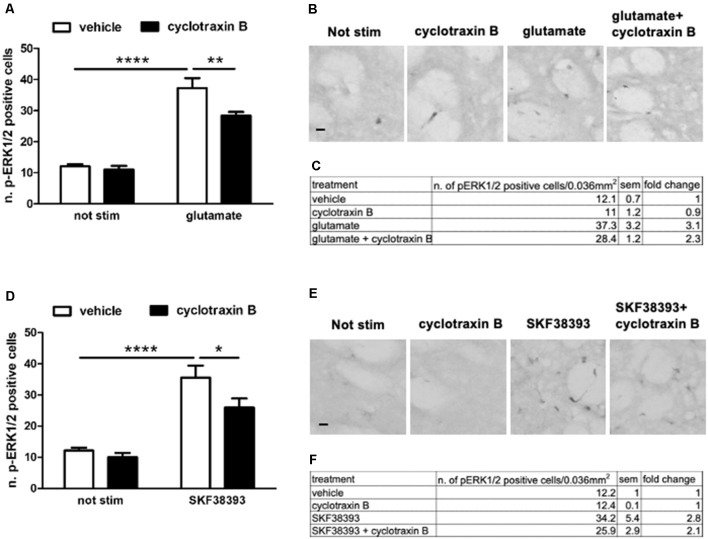
**(A,C)** Glutamate-induced ERK phosphorylation is significantly reduced by the pre-treatment with TrkB antagonist cyclotraxin B (two-way ANOVA, the effect of glutamate *F*_(1,28)_ = 135.165, *p* < 0.0001, the effect of cyclotraxin B *F*_(1,28)_ = 7.40, *p* = 0.0111, the effect of interaction *F*_(1,28)_ = 4.584, *p* = 0.04. Bonferroni’s *post hoc*, not stimulated vs. glutamate: ****p < 0.0001, glutamate vs. glutamate + cyclotraxin B: **p = 0.002). Data are shown as mean ± SEM, *n* = 8 per group. The values of mean, SEM, and fold change relative to vehicle are shown in panel **(C)**. **(B)** Representative pictures of anti-phospho-ERK1/2 immunohistochemistry. Scale bar: 50 μm. **(D,F)** Pre-treatment with cyclotraxin B downregulates SKF38393-induced ERK activation (two-way ANOVA, effect of SKF38393 *F*_(1,27)_ = 55.030, *p* < 0.0001, effect of cyclotraxin B *F*_(1,27)_ = 4.947, *p* = 0.035, effect of interaction *F*_(1,27)_ = 1.99, *p* = 0.17. Bonferroni’s *post hoc*, not stimulated vs. SKF38393 ****p < 0.0001, SKF38393 vs. SKF38393 + cyclotraxin B *p = 0.014). Data are shown as mean ± SEM. The values of mean, SEM, and fold change relative to not stimulated control are shown in panel **(F)**. Not stimulated: *n* = 8, cyclotraxin B: *n* = 7, SKF38393: *n* = 8, SKF38393 + cyclotraxin B: *n* = 8. **(E)** Representative pictures of anti-phospho-ERK1/2 immunohistochemistry. Scale bars: 50 μm.

To investigate this point, striatal slices were pre-treated for 1 h with 1 μM cyclotraxin B, a TrkB receptor antagonist, and then stimulated for 10 min with 10 μM glutamate or 10 μM SKF38393. As shown in Figure 6, both glutamate- and SKF38393-induced ERK phosphorylation was significantly downregulated, albeit not completely inhibited, upon TrkB blockade.

This result may suggest that BDNF-TrkB signaling is required for both NMDA/AMPA and D1 receptors to maximally induce ERK activation. However, this relationship is not reciprocal since BDNF-induced ERK activation is facilitated by the blockade of either D1 or NMDA/AMPA receptors.

## Discussion

In the present study, we preliminarily investigated the potential crosstalk between glutamatergic, dopaminergic, and BDNF inputs to ERK cascade in mouse striatal slices obtained from mature mouse brains. This approach allowed us to monitor ERK activation at the single-cell definition in a more physiological setting in comparison to other traditional techniques used to study signal transduction, such as the dissociated neuronal cultures and organotypic slices.

In recent years, the interplay between NMDARs and dopamine receptors has been extensively studied, given its essential physiological role but also its involvement in pathological conditions. It is now known that the ERK cascade is a common downstream target of glutamate and dopamine pathways in different brain regions, since its triggering requires both calcium influx from AMPAR/NMDAR and D1 receptors, but does not involve D2 receptors activation (Valjent et al., 2005; Kaphzan et al., 2006; Bertran-Gonzalez et al., 2008). Also, both NMDAR/AMPAR and D1R are potently activated during rewarding experience associated with addictive drugs and the signaling integration originated from these two neurotransmitter systems modulates the enduring synaptic changes underlying addictive behavior (Cahill et al., 2014b; Pascoli et al., 2014).

Our results, in agreement with previous findings obtained in organotypic slices (Fasano et al., 2009), further confirm that the combined stimulation of glutamate and D1 receptors has a synergistic effect on ERK phosphorylation. Also, we show that an intact D1R- mediated signaling is necessary for glutamate to activate ERK.

Interestingly, our study also suggests synergistic crosstalk between D1R- and BDNF-mediated signaling onto the ERK pathway. Previous work already indicated a link between D1 receptor-mediated activation of TrkB receptors and increased BDNF expression in striatal cultures. For instance, Iwakura et al. (2008) demonstrated that SKF39393 can transactivate TrkB receptors in striatal neurons and this action is accompanied by the phosphorylation of PLC_ϒ)_, Akt, and ERK. These findings were further extended by Williams and Undieh (2009) who showed that SKF38393, but not the D2 agonist quinpirole, increased BDNF protein expression in rat hippocampal and striatal slices. However, those studies cannot be directly reconciled with our findings. First of all, Iwakura et al. (2008) showed a D1R-mediated effect on the TrkB receptor after 3 h, while our study was limited to a short-term (10 min) effect. Similarly, Williams and Undieh (2009) showed an increased effect of SKF38393 on BDNF expression after 24 h treatment. More importantly, our data in Figure 5 indicate that BDNF-TrkB modulation of ERK signaling is constrained by D1R (and glutamate receptors) rather than enhanced. It is formally possible that this short-term inhibitory effect may mask a long-term positive modulation. However, our observation does represent the immediate biochemical interplay between BDNF and dopamine in striatal cells and should deserve some attention.

Importantly, the modulatory effect of D1 receptors on BDNF-TrkB-ERK could also be replicated with glutamate. In the presence of AMPAR and NMDAR antagonists, ERK phosphorylation is significantly enhanced in response to BDNF (see Figure 5). This is not entirely surprising since substantial evidence indicated that NMDARs and D1Rs do positively interact in striatal cells to stimulate ERK, also through physical interactions *via* GluN1 subunits and D1R-mediated phosphorylation of GluN2B (Pascoli et al., 2011; Cahill et al., 2014a). In that respect, D1Rs and NMDARs can be seen as an integrated system within striatal medium spiny neurons of the direct pathway able to activate the associated circuitry.

Also, our data show that the observed increase in ERK activation in response to the co-application of BDNF and either AMPAR/NMDAR and D1 antagonists does not lead to detectable a nuclear signaling enhancement, as measured as histone H3 phosphorylation. However, the assessment of a potential positive modulatory effect of glutamate and D1 receptor antagonists is technically challenging, considering that the dose we had to use to elicit detectable pH3 in response to BDNF (20 ng/ml) is already saturating, while the lower dose (10 ng/ml) did not activate pH3 at all, due to the non-graded dose-response for this marker.

An important additional consideration is related to the cell specificity of the observed modulation. TrkB and NMDA receptors are expressed in all mature MSNs, both in the direct and the indirect striatal pathways, while D1Rs are largely confined, in rodents, to the direct pathway. It is formally possible that the observed enhancement of BDNF-mediated ERK activation produced by the blockade of glutamate and D1 receptors occurs not in the dMSNs, where all three receptors are expressed, but rather in the MSNs of the indirect pathway (iMSNs). Alternatively, the observed enhancement of BDNF-mediated ERK phosphorylation may occur in dMSNs as a compensatory mechanism when the D1 and glutamate receptors activity is compromised. We currently have no clear hypothesis to explain this observation.

The complementary (and converse) experiment in Figure 6 aimed at determining whether TrkB receptors may modulate, directly or indirectly, D1 and glutamate receptors. The result, i.e., TrkB inhibition with cyclotraxin B attenuates both glutamate and SKF38393 ability to activate ERK signaling in MSNs, is certainly more in line with previous evidence indicating that BDNF signaling contributes to both dopamine and glutamate signaling.

The existence of cooperation between glutamate and BDNF is supported by an increasing number of studies. In this regard, it has been shown that BDNF enhances excitatory synaptic transmission in the hippocampus through pre- and post-synaptic mechanisms by enhancing glutamate release and regulating the expression, trafficking, and activity of both AMPA and NMDA receptors. For instance, in cultured hippocampal neurons activation of TrkB receptors by BDNF increases the delivery of NMDA receptors to the plasma membrane as well as the expression of the AMPAR subunits GluR1, GluR2, and GluR3 (Caldeira et al., 2007a,b). Interestingly, it has also been shown that BDNF-induced dendritic growth is mediated by ERK and requires both BDNF-induced CREB phosphorylation and the nuclear translocation of CREB-regulated transcription coactivator (CRTC1), triggered by NMDA receptors (Finsterwald et al., 2010). Similarly, signal integration mechanisms between BDNF and dopamine D1 receptors do also exist. For instance, in the hippocampus, dopamine-mediated persistence of long-term memory seems to be mediated by BDNF (Rossato et al., 2009). Furthermore, in the basolateral amygdala the threshold for LTP induction, which may be critically involved in the acquisition and consolidation of fear memory, is strictly dependent on the concurrent activation of postsynaptic D1 and TrkB receptors (Li et al., 2011).

Finally, the best indirect evidence available at the functional level in the striatum is the negative effect of TrkB inhibitors on acute amphetamine-dependent behavior (McGinty et al., 2010). However, once again, that effect seems rather indirect since amphetamine was able to increase TrkB phosphorylation only in a delayed manner, around 3 h post-injection, while ERK activation is already maximal as early as 15 min in response to psychostimulants (Valjent et al., 2005; Shi and McGinty, 2007).

Overall, our results support an unprecedented complexity in the crosstalk among glutamatergic, dopaminergic, and BDNF signaling converging onto the ERK pathway in the striatum. To be noted, our studies have been carried out only in male subjects so far. Further investigations will be necessary to clarify the biochemical details of how these signals integrate into the basal ganglia circuitry to modulate behavioral plasticity, also concerning possible sex differences.

## Data Availability Statement

The raw data supporting the conclusions of this article will be made available by the authors, without undue reservation.

## Ethics Statement

Ethical review and approval was not required for the animal study because all animals involved in this study did not undergo any regulated procedures but they were sacrificed by Schedule 1.

## Author Contributions

IM and HH performed the experiments. IM analyzed the data. IM and RB designed the experiments and wrote the manuscript. All authors contributed to the article and approved the submitted version.

## Conflict of Interest

The authors declare that the research was conducted in the absence of any commercial or financial relationships that could be construed as a potential conflict of interest.
